# Ni_1–*x*_Mn_*x*_Co_2_O_4_ Nanoparticles as High-Performance Electrochemical Sensor Materials for Acetaminophen Monitoring

**DOI:** 10.1021/acsomega.4c10927

**Published:** 2025-03-17

**Authors:** Alba Arenas-Hernandez, Francisco Enrique Cancino-Gordillo, Umapada Pal

**Affiliations:** 1 Institute of Physics, Autonomous University of Puebla, 18 Sur & Av. San Claudio, C.U., Puebla 72570, Mexico; 2 Instituto de Energias Renovables, Universidad Nacional Autonoma de Mexico, Priv. Xochicalco S/N, Temixco, Morelos 62580, Mexico

## Abstract

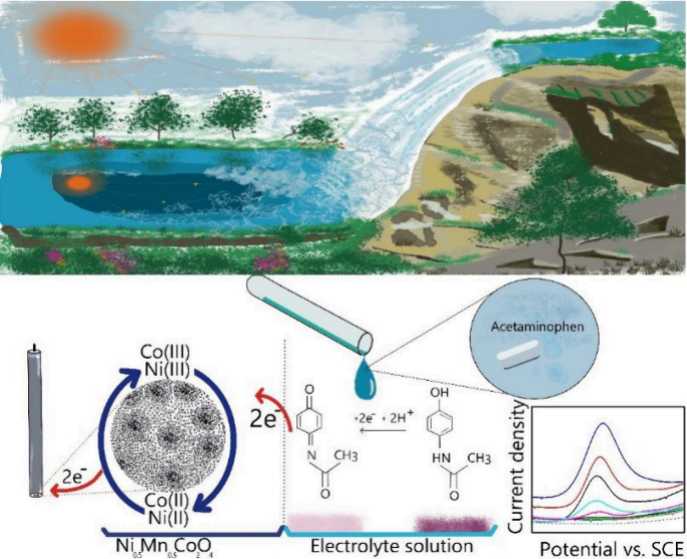

Ternary metal oxides, known for their superior electrical and optical properties compared to binary or conventional oxides, hold significant promise for catalysis and energy storage applications. This study investigates the electrochemical performance of Ni_1–*x*_Mn_*x*_Co_2_O_4_ nanoparticles for detecting acetaminophen in aqueous phosphate buffer solution. The cobaltite nanoparticles were obtained through a simple gel-combustion synthesis, and the sensors were characterized using cyclic voltammetry, chronoamperometry, and differential pulse voltammetry. The anodic peak currents associated with acetaminophen oxidation were assessed by varying the scan rate of current–voltage cycles. Among the sensors tested, the one fabricated with Ni_0.5_Mn_0.5_Co_2_O_4_ nanoparticles as an active material exhibited the highest sensitivity of 38.2 μA cm^–2^ mM^–1^ and a detection limit of approximately 2 μM, demonstrating its potential for sensitive and efficient acetaminophen detection. Moreover, the sensors fabricated using these ternary oxide nanostructures demonstrate a rapid chronoamperometric response time of 35.4 s and a decay lifetime of 0.31 s, highlighting the fast detection capabilities of acetaminophen. The electrochemical oxidation mechanism of acetaminophen and the charge transfer characteristics at the electrode–electrolyte interface have been discussed.

## 1 Introduction

Water pollution is one of the emerging problems at the current time, which has surged dramatically due to a range of contaminants, including dyes, heavy metals, viruses, bacteria, fungi, pesticides, microplastics, and pharmaceuticals. Many of these pollutants are inadequately monitored and poorly removed by conventional wastewater treatment processes.^1,2^ Pharmaceuticals, specifically developed to address human health issues, can pose significant risks to human health and ecosystems when their degradation or removal from water is inefficient.^1,2^ The presence of pharmaceuticals in water can lead to antibiotic resistance in bacteria, decreasing the efficacy of antibiotics in treating infections.^1^ Acetaminophen, caffeine, metronidazole, sulfamethoxazole, metformin, and naproxen are common pharmaceuticals found in elevated concentrations in water bodies worldwide.^1−3^

Acetaminophen (paracetamol), a popular analgesic and antipyretic, is one of the most frequently detected pharmaceuticals in contaminated water.^4^ As one of the most commonly consumed medicines worldwide, acetaminophen is frequently detected in surface and drinking water. Acetaminophen poses health risks due to hepatotoxicity caused by the formation of *N*-acetyl-*p*-benzoquinone imine during its oxidation, which has carcinogenic effects.^1,4^ Therefore, reliable detection of acetaminophen in water is essential for monitoring pollution levels and mitigating its environmental impact.

One effective way to monitor the presence of pharmaceuticals in water by using sensors. A variety of sensors, including biosensors, electronic sensors, microfluidic sensors, and electrochemical sensors, have been utilized for monitoring pollutants in water.^5,6^ While biosensors and electronic sensors have been used to monitor pollutants such as ammonium and arsenic ions in water effectively, electrochemical sensors have been utilized routinely to detect pharmaceuticals in water. Electrochemical sensors are particularly effective in pharmaceutical detection in aquatic environments due to their high sensitivity, selectivity, and rapid response time.^4^

The performance of electrochemical sensors depends mainly on the nature of the active material used to fabricate them. Nanostructures of metal oxides such as TiO_2_, ZnO, and ZrO_2_ have been commonly used to fabricate electrochemical sensors with superior performances.^3^ Bimetallic metal oxides such as NiFeO_*x*_ and NiCo_2_O_4_ have also been utilized for this purpose.^6,7^ The performance of bimetallic metal oxides is seen to be superior to monometallic metal oxides due to the multiple oxidation states of component metal ions and abundance of active electrocatalytic sites. For instance, while an electrochemical sensor fabricated with TiO_2_ nanorods achieved a sensitivity of 0.2 mA cm^–2^ mM^–1^ for glucose detection,^8^ the electrochemical sensors fabricated with Fe_*x*_Co_*y*_O_4_/rGO composite nanostructures^9^ and mesoporous ZnCo_2_O_4_ nanowires^10^ revealed sensitivities of 1.3 and 14 mA cm^–2^ mM^–1^ for the same application, respectively. The detection limits in the latter cases were also lower than in the earlier case. A sensor fabricated with the Fe_*x*_Co_*y*_O_4_/rGO composite achieved a detection limit as low as 0.07 mM and a sensitivity of 1.3 mA cm^–2^ mM^–1^.^9^ Meanwhile, the sensor fabricated with mesoporous ZnCo_2_O_4_ nanowires reached a sensitivity of 14 mA cm^–2^ mM^–1^ and a detection limit of 0.54 μM.^10^ While the good performance of the Fe_*x*_Co_*y*_O_4_/rGO composite-based sensor was associated with a higher number of active sites and excellent electrocatalytic behavior of the composite, the superior performance of the sensor fabricated with ZnCo_2_O_4_ nanowires has been attributed to a high concentration of oxygen vacancies at their surfaces.^10^

In the context of pollutant detection, Sudha et al.^11,12^ fabricated an electrochemical sensor using NiCo_2_O_4_ nanorods for the detection of carcinogenic hydrazine. The active material was prepared by a hydrothermal method, and the sensor achieved a sensitivity of 48.25 μA cm^–2^ mM^–1^ with a detection limit of 260 nM. The authors attributed this performance to the abundant electroactive sites of Ni and Co in the bimetallic oxide. Kumar et al.^12^ developed two electrochemical sensors for detecting paracetamol and dopamine using CoFe_2_O_4_ and MnFe_2_O_4_ nanoparticles as active materials. The nanostructures were prepared by a low-cost and simple combustion method. The CoFe_2_O_4_ and MnFe_2_O_4_ nanostructure-based sensors achieved sensitivities of 0.002 and 0.003 μA μM^–1^, with detection limits of 250 and 300 nM, respectively.^12^

To improve the performance of such electrochemical sensors in their sensitivity and detection limit, researchers have modified metal oxide nanostructure with different dopants, conjugation of metallic nanostructures, and carbonaceous materials to enhance charge carrier transport. For example, Pollap and collaborators developed a sensor to detect ciprofloxacin and acetaminophen using Au nanoparticle-conjugated TiO_2_ gel and mesoporous carbon.^13^ The sensor achieved a sensitivity of 15.93 μA μM^–1^ and a detection limit of 0.108 μM for ciprofloxacin, while its sensitivity and detection limit for acetaminophen were 11.56 μA μM^–1^ and 0.210 μM, respectively. In another study, Wang et al.^14^ fabricated an electrochemical sensor utilizing Co/CO_3_O_4_ composite nanoparticles and hollow nanoporous carbon polyhedrons, achieving a detection limit of 0.0083 μM and a sensitivity of 0.157 mA cm^–2^ μM^–1^ for acetaminophen. The composite material was prepared using a polydopamine/zeolitic imidazolate framework-67 template, followed by pyrolysis and oxidation processes. The improved acetaminophen detection performance of the sensor was attributed to multiple oxidation states of metal ions, ordered nanoporous carbon structure, and low metal oxide/electrolyte interface resistance. As can be observed, the sensitivity and detection limit of electrochemical sensors strongly depend on the electrochemical properties of the selected active material. For the detection of pharmaceuticals in water, the active electrocatalysts should have excellent sensitivity and low detection limits, along with low production costs. While NiCo_2_O_4_^11^ and MnCo_2_O_4_^15^ nanoparticles have been individually studied for various applications, comprehensive investigations into the effects of systematically varying the Mn content in Ni_1–*x*_Mn_*x*_Co_2_O_4_ nanoparticles,^16^ particularly for electrochemical sensor applications, are scarce. In this study, we fabricated electrochemical sensors using Ni_1–*x*_Mn_*x*_Co_2_O_4_ nanoparticles for three different values of *x* (*x* = 0, 0.5, and 1.0), as the active material for paracetamol detection. The nanostructures were synthesized by a simple and low-cost gel-combustion method and characterized using X-ray diffraction (XRD), scanning electron microscopy (SEM), transmission electron microscopy (TEM), and N_2_ adsorption–desorption studies. Electrochemical properties of the nanostructures were evaluated through electrochemical impedance spectroscopy (EIS) and cyclic voltammetry (CV) characterizations. Electrochemical sensors fabricated using the nanostructures were tested by cyclic voltammetry (CV), differential pulse voltammetry (DPV), and chronoamperometry techniques.

## 2 Experimental Section

### 2.1 Reagents

Nickel nitrate hexahydrate (Ni(NO_3_)_2_·6H_2_O, Fermont), manganese nitrate tetrahydrate (Mn(NO_3_)_2_·4H_2_O, Sigma-Aldrich), cobalt nitrate hexahydrate (Co(NO_3_)_2_·6H_2_O, Fermont), polyvinylpyrrolidone (PVP, *M*_w_ = 40,000; Sigma-Aldrich), and glycine (H_2_NCH_2_COOH, Sigma-Aldrich) were employed as received for the synthesis of the active material of the electrochemical sensor. Phosphate-buffered saline (PBS, pH 7, Meyer), acetaminophen (C_8_H_9_NO_2_, Sigma-Aldrich), acetic acid (CH_3_CO_2_H, Sigma-Aldrich), and sodium hydroxide (NaOH, Sigma-Aldrich) were utilized for the preparation of the electrolytic solution. Deionized (DI) water from a Millipore water purification equipment (ρ > 18.2 ΜΩ cm) was used for cleaning glassware and washing the sample and electrodes.

### 2.2 Synthesis of Ni_1–*x*_Mn_*x*_Co_2_O_4_ NPs

**Figure 1:**
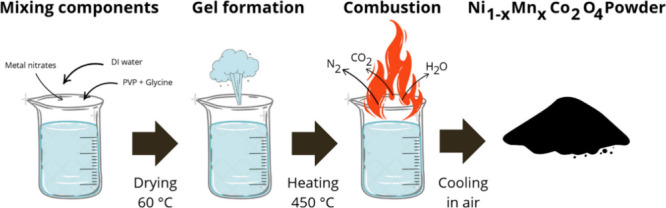
Schematic presentation of the synthesis process used for obtaining Ni_1–*x*_Mn_*x*_Co_2_O_4_ nanoparticles.

For the synthesis of Ni_1–*x*_Mn_*x*_Co_2_O_4_ (NMCO) nanoparticles with three different Mn molar fractions (*x* = 0.00, 0.50, and 1.00), a solution was prepared by dissolving 1 – *x* mmol of nickel nitrate, *x* mmol of manganese nitrate, and 2 mmol of cobalt nitrate in 20 mL of DI water under continuous magnetic stirring for 30 min. Subsequently, 6.4 g of polyvinylpyrrolidone (PVP, *M*_w_ = 40,000) was slowly added to the solution, with stirring extended for an additional 50 min to ensure the formation of a homogeneous, transparent solution. Next, 375 mg of glycine, serving as fuel,^16,17^ was introduced and mixed into the solution with further magnetic stirring for 30 min. The resulting homogeneous solution was transferred to a porcelain combustion boat and heated on a hot plate at 60 °C overnight to ensure full solvent evaporation. The dried gel in the ceramic boat was then heated to 450 °C in a horizontal furnace (Carbolite) at a 3 °C/min heating rate for 6 h. The solid cobaltite was formed, and several gases, such as CO_2_, NO_*x*_, and N_2_ were released on ignition. After cooling to room temperature, the product was collected, ground in an agate mortar, and stored in a glass vial for further use. A schematic of the synthesis process is shown in Figure 1. To adjust the molar ratio of Ni and Mn in the Ni_1–*x*_Mn_*x*_Co_2_O_4_ nanoparticles, a specific molar fraction of the Ni precursor was replaced with the Mn precursor in the reaction mixture while keeping all other synthesis parameters the same. Using this approach, three distinct samples were obtained: NiCo_2_O_4_ (*x* = 0.0), Ni_0.5_Mn_0.5_Co_2_O_4_ (*x* = 0.5), and MnCo_2_O_4_ (*x* = 1.0). These samples were subsequently characterized to evaluate their structural, morphological, and compositional properties. Furthermore, the nanoparticles were tested for potential applications in sensor devices.

### 2.3 Characterization of Ni_1–*x*_Mn_*x*_Co_2_O_4_ Nanoparticles

The morphology of the nanoparticles was examined in a JEOL JSM-7800F field-emission scanning electron microscope (FESEM) operating at 5.0 kV. The elemental composition of the nanostructures was analyzed by energy-dispersive X-ray spectroscopy (EDS) with an X-Max spectrometer (Oxford Instrument), using an acceleration voltage of 15 kV in the FESEM. Samples for FESEM and EDS characterizations were prepared by depositing the nanoparticles’ colloidal suspension (in ethanol) onto silicon wafers and then drying at room temperature. More detailed morphological and fine-structure analyses were performed using high-resolution transmission electron microscopy (HRTEM). HRTEM images of the samples were obtained in a JEOL 2100F microscope operating at 200 kV. The samples for TEM and HRTEM observation were prepared by dispersing the nanostructures onto carbon-coated copper grids by drop-casting their colloidal suspension in ethanol, followed by drying at room temperature. X-ray diffraction (XRD) patterns of the NMCO nanoparticles were obtained using CuKα_1_ radiation (λ = 1.5406 Å) on a Panalytical Empyrean diffractometer at room temperature, employing a low-background silicon sample holder. Data were collected in the 2θ range of 15–90°, with a step size of 0.02°. The average pore size and specific surface area are important parameters in fabricating electrochemical sensors. A larger surface area can increase the number of available active sites in the sensor. To evaluate these characteristics, we measured nitrogen adsorption–desorption isotherms of the nanostructures at 77 K using a BELSORP Mini-II system (BEL, Japan) texture analyzer. Before adsorption–desorption measurements, the samples were degassed at 150 °C under 10 Pa vacuum for 18 h.

### 2.4 Electrode Preparation

The working electrodes, Ni_1–*x*_Mn_*x*_Co_2_O_4_/CE, were prepared by a drop-dry method. Before the deposition of active materials, the Teflon-coated carbon electrode (CE, CORRTEST) was polished with alumina powder slurries (0.25 μm) on a polishing pad and cleaned multiple times with DI water. To prepare the active material paste, 0.75 mL of DI water and 0.25 mL of methanol were mixed. Subsequently, 10 mg of Ni_1–*x*_Mn_*x*_Co_2_O_4_ nanoparticles and 0.1 mL of 5 wt % Nafion solution were added to the mixture under magnetic stirring for 2 h at room temperature. A 12 μL aliquot of this solution was dropped onto the CE. The electrode was dried at room temperature for 4 h, followed by additional drying at 70 °C for 4 h in a gravity furnace.

### 2.5 Electrochemical Characterization of Ni_1–*x*_Mn_*x*_Co_2_O_4_ Nanoparticles

CV was employed to monitor the oxidation and reduction process of acetaminophen in an electrolyte solution containing 10 mM acetaminophen in 0.1 mM PBS (adjusted to pH 6.5), under a scan rate of 10 mV/s. A CE (3 mm diameter) or Ni_1–*x*_Mn_*x*_Co_2_O_4_-covered CE was used as the working electrode, while a saturated calomel electrode (SCE) and platinum foil were used as the reference electrode and counter electrode, respectively. For the CV experiments, the window potential was swept between −0.1 and 0.8 V with respect to the SCE. The CV measurements were conducted using a CORRTEST CS2350 potentiostat, with a 2 cm separation between the working and counter electrodes. To determine the diffusion constant of acetaminophen on the Ni_1–*x*_Mn_*x*_Co_2_O_4_/CE electrode, the electrolyte solution containing 5 mM acetaminophen in 0.1 mM PBS (pH 6.5) was used, and the scan rate was varied between 5 and 75 mV/s.

Differential pulse voltammetry (DPV) was employed to determine the sensitivity of the fabricated sensors by analyzing them in various concentrations of acetaminophen ranging from 0.01 to 5 mM, in 0.1 M PBS of pH 6.5. The DVP measurements were carried out with an initial voltage of 0.3 V, a final voltage of 0.9 V, an amplitude of 0.025 V, a pulse width of 0.01 s, and a pulse period of 0.5 s for all experiments.

EIS was used to study the electron transfer kinetics of the active materials. EIS measurements were performed in a solution of 5 mM acetaminophen in 0.1 M PBS (pH 6.5) over a frequency range of 1 MHz to 0.1 Hz, measuring a potential of 0.4 V and an amplitude of 50 mV. Chronoamperometry was performed to estimate the characteristic decay lifetime constants. The measurements were conducted with an initial voltage of 0.0 V relative to the SCE and a second voltage of 0.4 V for a duration of 100 s across all experiments. The target solution was 5 mM acetaminophen in 0.1 M PBS (pH 6.5).

## 3 Results and Discussion

**Figure 2:**
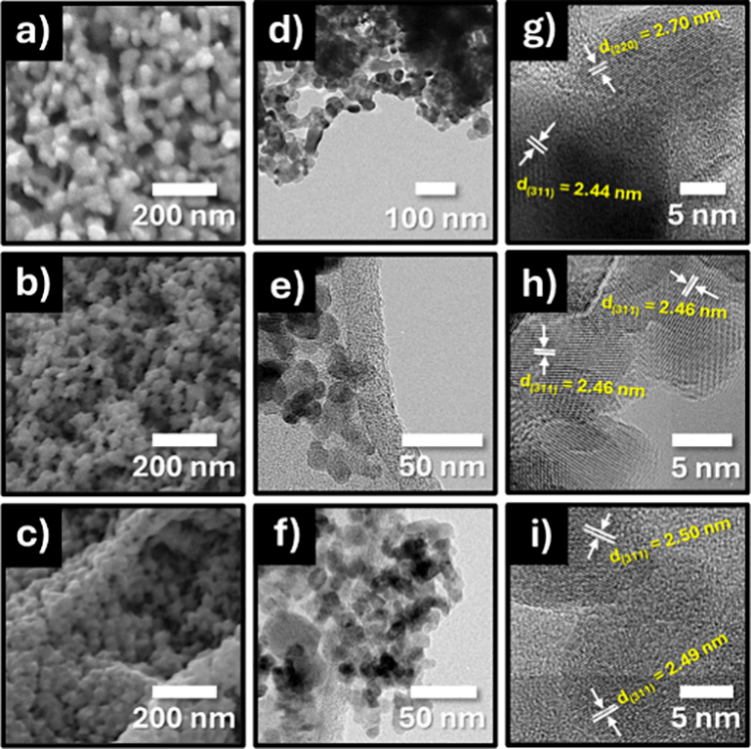
Typical FESEM images of the (a) NiCo_2_O_4_, (b) Ni_0.5_Mn_0.5_Co_2_O_4_, and (c) MnCo_2_O_4_ nanoparticles; low- and high-resolution TEM micrographs of the (d, g) NiCo_2_O_4_, (e, h) Ni_0.5_Mn_0.5_Co_2_O_4_, and (f, i) MnCo_2_O_4_ nanoparticles.

The morphologies of the synthesized MnCo_2_O_4_, NiCo_2_O_4_, and Ni_0.5_Mn_0.5_Co_2_O_4_ nanoparticles were analyzed using FESEM. Figure 2a–c presents typical FESEM images, revealing that all three materials consist of quasi-spherical particles with less than 100 nm in size and weakly agglomerated. These small agglomerates are owing to the uniform heating of the reaction mixture that took place in the combustion,^18^ which can be considered as loosely packed porous structures. The FESEM micrographs for the MnCo_2_O_4_ sample revealed pores and voids in the compound, which can be attributed to the large amount of gases escaping from the reaction mixture during combustion compared with the other samples. The size of the agglomerates can influence morphological and textural properties such as the surface area and pore size.^19^ For a more comprehensive analysis of morphology, size, and microstructure and to investigate the impact of Mn ion substitution with Ni ions, the nanoparticles were examined through TEM and HRTEM. Figure 2d–f displays typical TEM images of MnCo_2_O_4_, NiCo_2_O_4_, and Ni_0.5_Mn_0.5_Co_2_O_4_ nanoparticles, all displaying consistent quasi-spherical morphologies. The HRTEM images (Figure 2g–i) further confirm the high crystallinity of the nanoparticles, evidenced by the well-resolved atomic planes. Measurements of individual particles indicated an average size of 25.8 ± 6.1 nm for NiCo_2_O_4_ particles. As the Mn content increases, the average particle size significantly decreases to 9.5 ± 1.7 nm for MnCo_2_O_4_, illustrating the effect of Mn incorporation on particle size reduction. For an equal fraction of Ni and Mn ions, the average size of the nanoparticles is 10.4 ± 2.8 nm (Figure S1, Supporting Information). As can be noted, when the Ni ions are substituted by Mn ions, the interplanar spacing of the principal (311) lattice planes increases from 2.44 Å for NiCo_2_O_4_ to 2.49 Å for MnCo_2_O_4_, indicating a lattice expansion due to the substitution of the Co^3+^ and Ni^2+^ cations located at tetrahedral and octahedral sites by Co^2+^ and Mn^3+^ cations. The ionic radii of Co^2+^, Mn^3+^, Ni^2+^, and Co^3+^ are 0.745, 0.645, 0.69, and 0.545 Å, respectively.^20,21^ In the case of Ni_0.5_Mn_0.5_Co_2_O_4_, the interplanar spacing (2.46 Å) is similar to that of NiCo_2_O_4_ and smaller than that of MnCo_2_O_4_. Changes in the interplanar spacing can affect the electrical properties, particularly the charge carrier transport in metal oxide/electrolyte interfaces for electrochemical devices, as noted by several research groups.^22,23^

**Figure 3:**
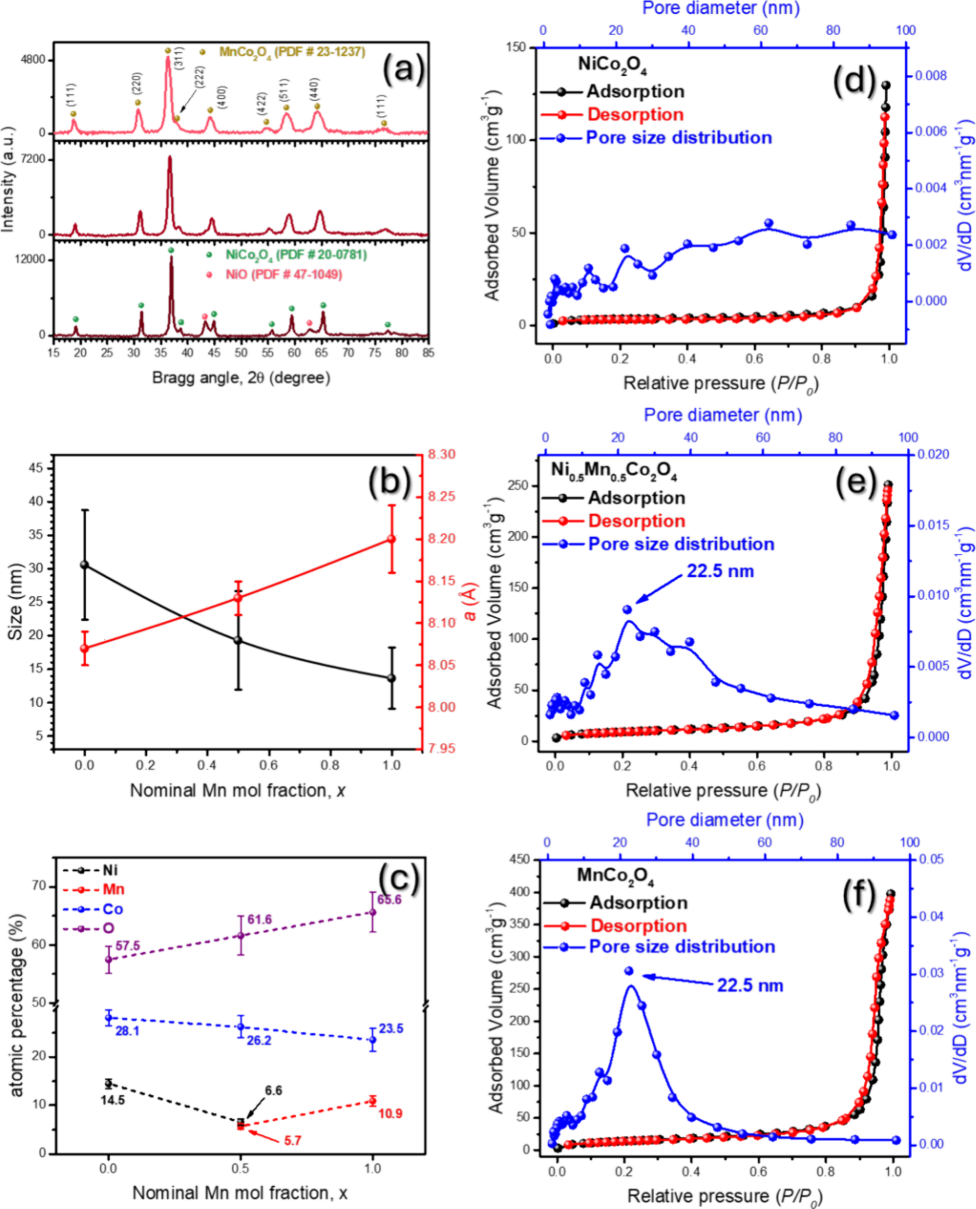
(a) XRD patterns of Ni_0.5_Mn_0.5_Co_2_O_4_, NiCo_2_O_4_, and MnCo_2_O_4_ nanoparticles. (b) Lattice constant and average grain size obtained from the Scherrer equation, as a function of the Mn incorporation. (c) EDS-estimated elemental composition of the synthesized nanoparticles. N_2_ adsorption–desorption isotherms and pore distribution in the (d) NiCo_2_O_4_, (e) Ni_0.5_Mn_0.5_Co_2_O_4_, and (f) MnCo_2_O_4_ samples.

Powder XRD patterns of the samples were recorded to assess their purity, crystal structure, and structural parameters (Figure 3a). The XRD patterns were compared with standard diffraction files for NiCo_2_O_4_ (JCPDS no. 20-0781), NiO (JCPDS no. 47-1049), and MnCo_2_O_4_ (JCPDS no. 23-1237).^24−26^ The average grain size was estimated using the Scherrer equation applied to three significant diffraction peaks, yielding average sizes of 35.8 nm for NiCo_2_O_4_, 14.1 nm for Ni_0.5_Co_0.5_O_4_, and 11.0 nm for MnCo_2_O_4_. Corresponding lattice parameters (*d*_311_) were 8.09, 8.18, and 8.20 Å, respectively (Figure 3b). Previous studies suggested that Ni_1–*x*_Mn_*x*_Co_2_O_4_ materials possess a spinel cubic structure, indicating that the substitution of Ni ions with Mn ions results in a decrease in grain size and an increase in lattice parameter “*a*”.^16,24,25^ The lattice constant reaches its maximum at full Mn incorporation (*x* = 1.0), while the grain size decreases correspondingly. This lattice expansion is attributed to the substitution of Co^2+^ and Mn^3+^ cations occupying tetrahedral and octahedral sites, respectively, in place of Co^3+^ and Ni^2+^ ions.^25^ The larger ionic radii of Co^2+^ (0.745 Å) compared to Co^3+^ (0.545 Å) and the small difference between the ionic radii of Mn^3+^ (0.645 Å) and Ni^2+^ (0.69 Å) facilitate this structural change, as evidenced by the consistent increase in the lattice parameter, which is consistent with results observed in HRTEM (Figure 2g–i). This is further illustrated by the shift of the principal diffraction peak for the three materials (Figure S2, Supporting Information), which gradually moved to lower angles as Ni is substituted for Mn, indicating lattice expansion. In the NiCo_2_O_4_ sample, two distinct peaks at 43.26 and 62.90° were observed, corresponding to the NaCl-type cubic structure of Ni_1–*x*_Co_*x*_O. EDS-estimated elemental compositions of the MnCo_2_O_4_, NiCo_2_O_4_, and Ni_0.5_Mn_0.5_Co_2_O_4_ nanoparticles are presented in Figure 3c and Table S1 (Supporting Information). The ratios of (Ni + Mn)/Co calculated were found to be 0.46, 0.47, and 0.52 for MnCo_2_O_4_, Ni_0.5_Mn_0.5_Co_2_O_4_, and NiCo_2_O_4_ samples, respectively, indicating that the materials are close to their expected stoichiometric compositions (1:2 ratio). The absence of additional peaks associated with secondary phases such as NiO and the closeness with the ideal ratio composition of (Ni + Mn)/Co confirm the phase purity of the material.^16,24,25^

Additionally, we analyzed the N_2_ adsorption–desorption isotherms and the pore size distributions for the MnCo_2_O_4_, Ni_0.5_Mn_0.5_Co_2_O_4_, and NiCo_2_O_4_ nanoparticles at 77 K, as shown in Figure 3d–f. The BET surface area, pore diameter, and pore volume were estimated using the Barrett–Joyner–Halenda (BJH) method. Both Ni_0.5_Mn_0.5_Co_2_O_4_ and MnCo_2_O_4_ samples exhibited type-IV isotherms with hysteresis loops characteristic of mesoporous structures.^27^ These hysteresis loops, indicative of capillary condensation within mesopores, were particularly evident at relative pressures close to *p*/*p*_0_ = 0.86, suggesting the onset of capillary condensation within the agglomerate voids. The pore distribution curves for Ni_0.5_Mn_0.5_Co_2_O_4_ and MnCo_2_O_4_ samples show well-defined peaks around 22.5 nm, confirming their mesoporous nature. In contrast, the NiCo_2_O_4_ sample displayed a type-II isotherm, typical of macroporous materials, with no well-defined peak in the pore size distribution curve, suggesting the formation of a hierarchical pore structure.^28^ Furthermore, the BET analysis revealed a significant increase in the specific surface area with increasing Mn content, from 12.32 m^2^ g^–1^ for NiCo_2_O_4_ to 33.71 m^2^ g^–1^ for Ni_0.5_Mn_0.5_Co_2_O_4_ and further to 53.22 m^2^ g^–1^ for MnCo_2_O_4_. This increase can be attributed to the reduction in the average size of the particles with Mn substitution observed in Figure 2. Additionally, the pore volume increased with the substitution of Ni ions by Mn ions in the lattice, with values of 0.18, 0.38, and 0.59 cm^3^ g^–1^ for NiCo_2_O_4_, Ni_0.5_Mn_0.5_Co_2_O_4_, and MnCo_2_O_4_, respectively.

**Figure 4:**
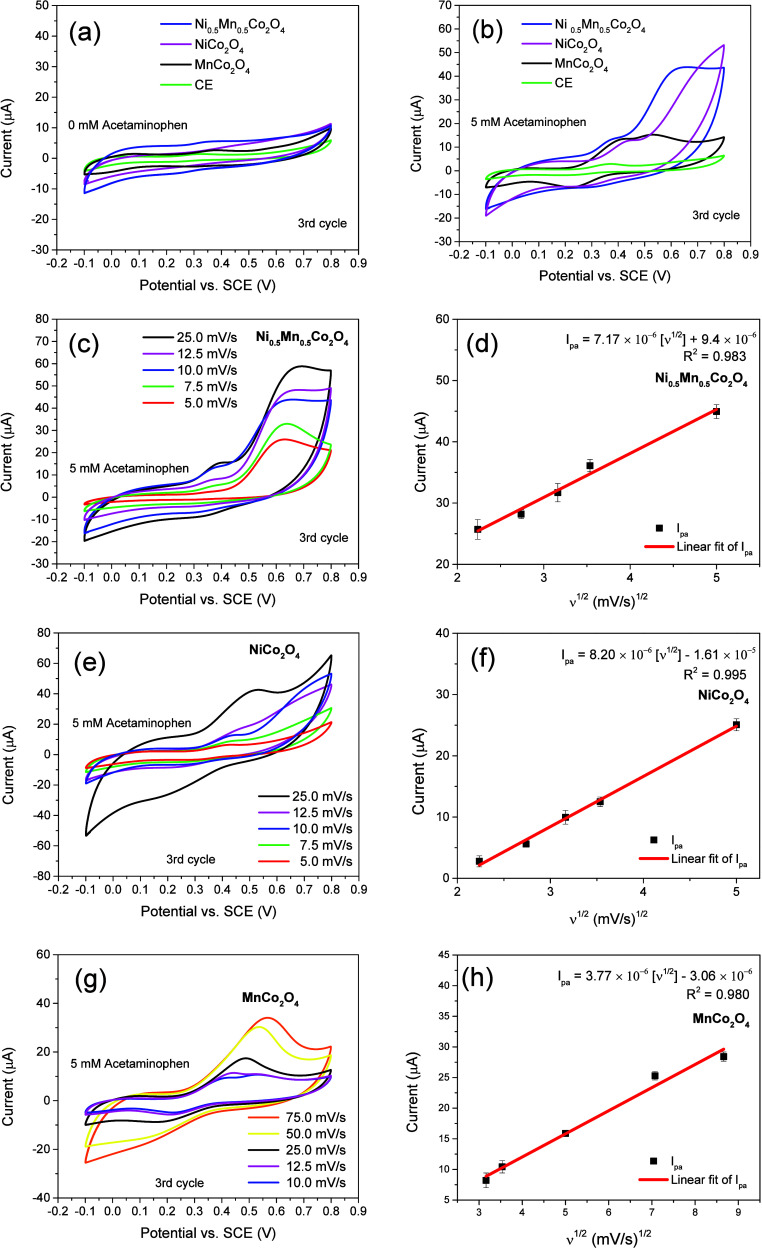
(a) Cyclic voltammograms of different active materials and (b) voltammograms of different active materials in 5 mM acetaminophen solution and a scan rate of 10 mV/s. Cyclic voltammograms of (c) Ni_0.5_Mn_0.5_Co_2_O_4_, (e) NiCo_2_O_4_, and (g) MnCo_2_O_4_ sensors at different scan rates and (d, f, h) corresponding *I*_pa_ vs *v*^1/2^ plots.

Figure 4a presents the voltammograms of Ni_0.5_Mn_0.5_Co_2_O_4_, NiCo_2_O_4_, and MnCo_2_O_4_ electrodes, measured using 0.1 M PBS adjusted to pH 6.5 without acetaminophen, which showed no oxidation or reduction peaks. On the other hand, Figure 4b illustrates the voltammograms of the same electrodes recorded using 5 mM acetaminophen solutions, revealing a notable oxidation peak across all materials. Among the electrodes, Ni_0.5_Mn_0.5_Co_2_O_4_ achieved the highest oxidation peak current (at 0.64 V), indicating the superior catalytic activity for acetaminophen electro-oxidation. Additionally, the absence of a reduction peak suggests that the anodic current peak potential (*E*_pa_) is conducive to the electro-oxidation of acetaminophen, and the process is irreversible.^4,29,30^

The effect of the potential scan rate on the electrocatalytic response of the fabricated electrodes was examined through cyclic voltammetry (Figure 4c,e,g) in the 5–25 mV/s scan rate range for a constant acetaminophen concentration of 5 mM in 0.1 M PBS (pH 6.5) solution. At a scan rate of 10 mV/s, the anodic current peaks appeared at 0.64 V for Ni_0.5_Mn_0.5_Co_2_O_4_ and 0.45 V for NiCo_2_O_4_ electrodes, whereas the anodic current peak was located at 0.41 V for the MnCo_2_O_4_ electrode. As the scan rate increased, the anodic peaks shifted to more positive potentials. A summary of *I*_pa_, *E*_pa_, and scan rates for the Ni_0.5_Mn_0.5_Co_2_O_4_ electrode is provided in Table 1. Corresponding data for the NiCo_2_O_4_ and MnCo_2_O_4_ electrodes are presented in Table S2 (Supporting Information).

**Table 1:** Summary of Peak Current (*I*_pa_) and Peak Potential (*E*_pa_) Values Obtained from CV Curves for the Ni_0.5_Mn_0.5_Co_2_O_4_ Electrode in the Detection of 5 mM Acetaminophen at Various Scan Rates

**electrode material**	**scan rate** (*v***)**	***I***_**pa**_	***E***_**pa**_
Ni_0.5_Mn_0.5_Co_2_O_4_	25.0 mV/s	4.49 × 10^–5^ A	0.69 V
	12.5 mV/s	3.61 × 10^–5^ A	0.67 V
	10.0 mV/s	3.17 × 10^–5^ A	0.64 V
	7.5 mV/s	2.82 × 10^–5^ A	0.63 V
	5.0 mV/s	2.57 × 10^–5^ A	0.62 V

Figure 4d,f,h presents the variations of anodic peak current intensity (*I*_pa_) with the square root of the scan rate (*v*^1/2^) for the three electrodes, showing a linear increase in *I*_pa_ with an increasing scan rate.^31^ The linear relations between the *I*_pa_ and *v*^1/2^ for the three sensors made of Ni_0.5_Mn_0.5_Co_2_O_4_, NiCo_2_O_4_, and MnCo_2_O_4_ followed the linear relations expressed by eqs 1, 2, and 3, respectively:
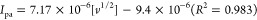
1
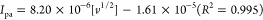
2
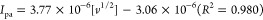
3

The high correlation coefficient (*R*^2^) obtained for each fit confirms a good linear relationship. The linear relations between *I*_pa_ and *v*^1/2^ indicate that the redox reactions (acetaminophen oxidation) occurring at the surface of the electrodes made of cobaltite nanostructures are typically diffusion-controlled, as suggested by Wang et al.^14^

To calculate the diffusion coefficient of acetaminophen on the electrode surfaces, we applied the Randles–Sevcik equation,^29^ which relates *I*_pa_ with the square root of the diffusion coefficient and scan rate as follows (eq 4):
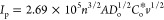
4where *n* is the number of electrons transferred in the redox reaction, *A* (cm^2^) is the electrode active area, *D*_o_ (cm^2^s^–1^) is the diffusion coefficient of the oxidized analyte, and  is the bulk concentration of the analyte (mol cm^–3^). Using *n* = 2, *A* = 0.07 cm^–2^, and  = 5 × 10^–3^ M, the values of *D*_o_ were estimated around 7.24 × 10^–10^, 9.48 × 10^–10^, and 2.00 × 10^–10^ cm^2^/s for the Ni_0.5_Mn_0.5_Co_2_O_4_, NiCo_2_O_4_, and MnCo_2_O_4_ electrodes, respectively, demonstrating that the NiCo_2_O_4_ sensor has the fastest *D*_o_ of species compared with other sensors.

**Figure 5:**
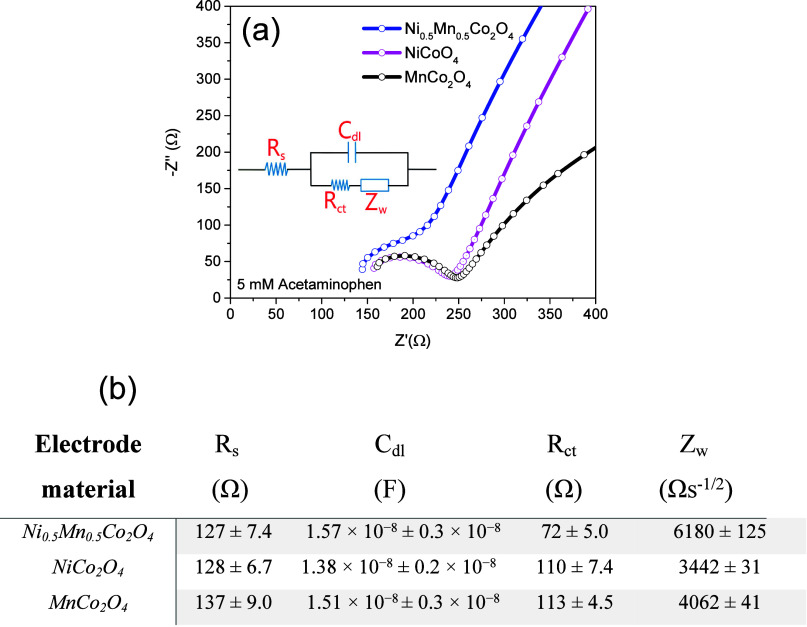
(a) EIS spectra for 5 mM acetaminophen using Ni_0.5_Mn_0.5_Co_2_O_4_, NiCo_2_O_4_, and MnCo_2_O_4_ as active materials in the electrochemical sensor, along with the corresponding equivalent circuit model. (b) Modeling parameters extracted from the Nyquist plots for acetaminophen detection using different active materials.

Electrochemical impedance spectroscopy (EIS) was employed to assess the electrochemical properties of the materials used in sensor fabrication. Figure 5a presents the Nyquist plots for NiCo_2_O_4_, Ni_0.5_Mn_0.5_Co_2_O_4_, and MnCo_2_O_4_ nanoparticles, used as active materials in the fabricated sensors. The inset shows the corresponding equivalent circuit model, based on a Randles circuit, that describes the electrochemical behavior of the system. In this model, *R*_s_ represents the solution resistance in Ω, *R*_ct_ denotes the charge transfer resistance in Ω, *C*_dl_ is the double-layer capacitance in Farad, and *Z*_w_ corresponds to the Warburg impedance in Ω s^–1/2^.^15,32^ Notable differences are observed in the Nyquist plots for the NiCo_2_O_4_, Ni_0.5_Mn_0.5_Co_2_O_4_, and MnCo_2_O_4_ sensors. Specifically, the Ni_0.5_Mn_0.5_Co_2_O_4_ electrode exhibits a smaller semicircular arc, indicating a lower *R*_ct_ and enhanced charge transfer kinetics.^33^ However, the semicircle for this electrode (blue curve) is incomplete at the higher frequencies, suggesting that the arc transitions into a linear to sinusoidal behavior. This incomplete arc indicates faster charge transfer at the electrode/electrolyte interface compared to the NiCo_2_O_4_ and MnCo_2_O_4_ electrodes, which is consistent with a higher *C*_dl_ for the Ni_0.5_Mn_0.5_Co_2_O_4_ electrode.^33^ These findings suggest that the Ni_0.5_Mn_0.5_Co_2_O_4_ electrode possesses superior electrochemical properties, particularly in terms of faster charge transport at the electrode surface.^34^

The modeling parameters presented in Figure 5b further confirm that the Ni_0.5_Mn_0.5_Co_2_O_4_-based sensor exhibits the lowest *R*_ct_, indicating enhanced charge transfer at the metal oxide/electrolyte interface. Such improved electrochemical behavior of the electrode can be attributed to the synergistic redox pair transitions of Co(III)/Co(II), Ni(III)/Ni(II), and Mn(III)/Mn(II),^14^ along with the shorter interplanar distance of the (311) planes in the spinel structure. Additionally, the *C*_dl_ parameter reflects two capacitances in series: *C*_st_, representing the capacitance of the Stern layer, and *C*_d_, representing the capacitance of the Gouy–Chapman layer.^35^ The higher *C*_dl_ observed for the Ni_0.5_Mn_0.5_Co_2_O_4_-based sensor is associated with a thinner Stern layer, which facilitates efficient charge transfer at the metal oxide/electrolyte interface.

**Figure 6:**
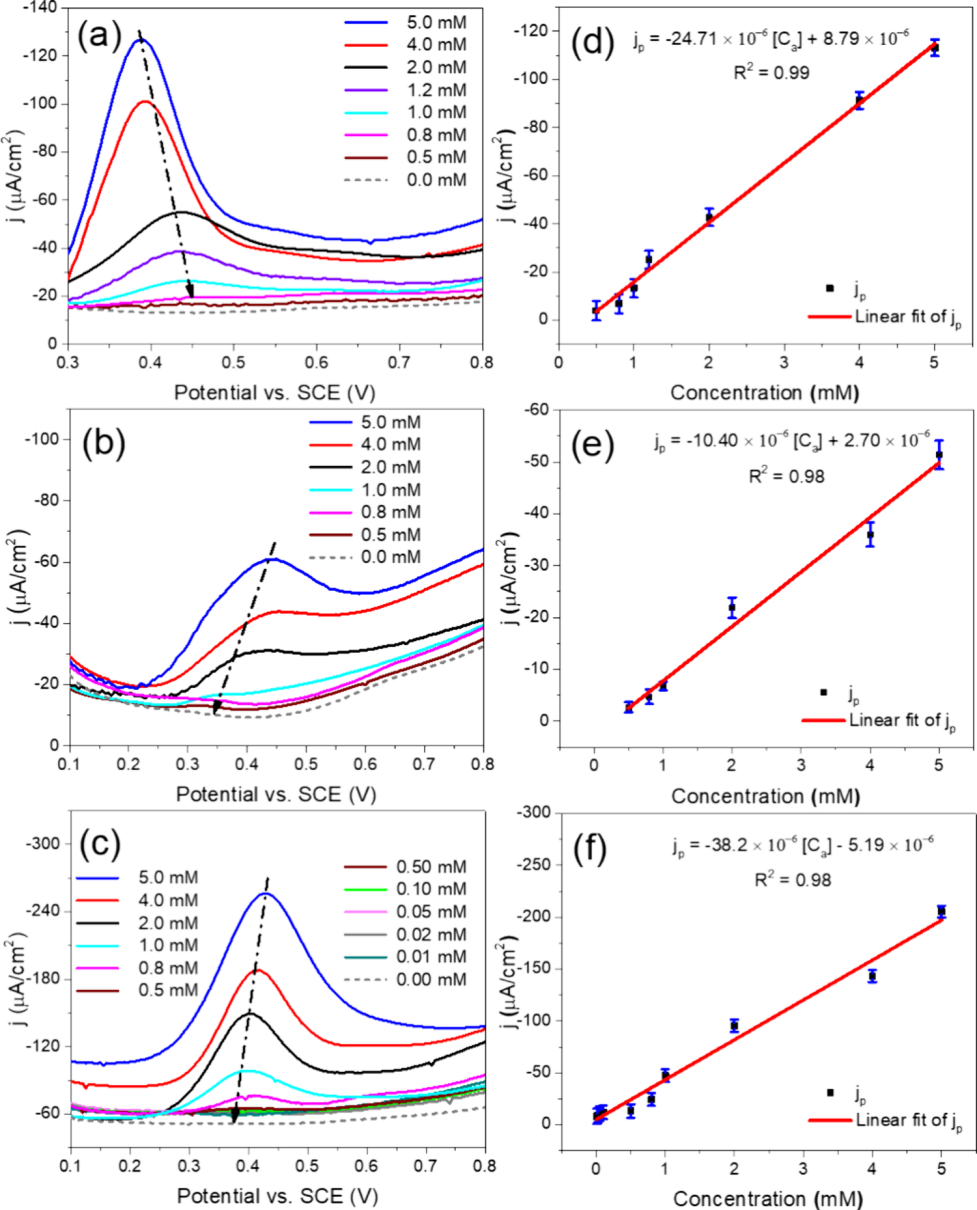
Differential pulse voltammetry response curves for varying acetaminophen concentrations using (a) NiCo_2_O_4_, (b) MnCo_2_O_4_, and (c) Ni_0.5_Mn_0.5_Co_2_O_4_ as the active materials in the electrochemical sensor and (d, f, and g) corresponding calibration curves of peak current versus acetaminophen concentration.

Figure 6 displays the DPV response curves for NiCo_2_O_4_, Ni_0.5_Mn_0.5_Co_2_O_4_, and MnCo_2_O_4_ nanoparticles, which were used as active materials for acetaminophen detection across the concentration range of 0.01–5.0 mM. For the NiCo_2_O_4_ sensor (Figure 6a), the oxidation peak appeared at approximately 0.38 V when the acetaminophen concentration was 5.0 mM. As the acetaminophen concentration decreased, the intensity of the oxidation peak decreased, along with a shift in its position to more positive potential. The lowest detectable acetaminophen concentration for this sensor was 0.5 mM. In contrast, the MnCo_2_O_4_ sensor revealed (Figure 6b) an oxidation peak at around 0.45 V for the acetaminophen concentration of 5.0 mM. While the intensity of this peak decreased with the reduction of acetaminophen concentration, the peak position shifted toward a more negative potential, in contrast to the behavior observed with the NiCo_2_O_4_ sensor.

Finally, the sensor prepared with Ni_0.5_Mn_0.5_Co_2_O_4_ nanoparticles (Figure 6c) revealed an oxidation peak around 0.43 V for 5.0 mM acetaminophen. This sensor exhibited the best electrochemical performance, demonstrating exceptional sensitivity for acetaminophen detection, with the lowest detected acetaminophen concentration of 0.01 mM. The current density for this sensor was approximately four times higher than that for the MnCo_2_O_4_ sensor and about 1.8 times higher than for the NiCo_2_O_4_ sensor. Similar to the MnCo_2_O_4_ sensor, a decrease in acetaminophen concentration resulted in a reduction in oxidation peak intensity and a shift toward the negative potential. The EIS results (Figure 5b) indicate that the Ni_0.5_Mn_0.5_Co_2_O_4_ sensor exhibits the highest capacitance, followed by the MnCo_2_O_4_ sensor. The observed shift of the oxidation peak to lower potentials with the decrease in acetaminophen concentration suggests a reduction in the energy barrier for electron transfer, likely due to a thinner Stern layer. This, in turn, facilitates more efficient diffusion of chemical species at lower concentrations.^35^

Figure 6d–f shows the calibration curves correlating current density with acetaminophen concentration, ranging from 0.01 to 5.0 mM, for each of the electrochemical sensors. All three sensors exhibited excellent linearity, with the following equations (eqs 5–7) representing the relationship between peak current density (*j*_p_) and acetaminophen concentration (*C*_a_):
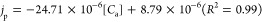
5
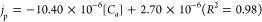
6
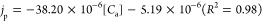
7

The sensitivity values for acetaminophen detection were calculated for the NiCo_2_O_4_, Ni_0.5_Mn_0.5_Co_2_O_4_, and MnCo_2_O_4_ sensors, yielding values of 24.71, 38.2, and 10.40 μA cm^–2^ mM^–1^, respectively. The limit of detection (LOD) was determined using the formula LOD = 3*s*/*m*, where *s* is the standard deviation of the blank measurement and *m* is the slope of the calibration curve.^36^ The *s* values of the blank measurements for the NiCo_2_O_4_, Ni_0.5_Mn_0.5_Co_2_O_4_, and MnCo_2_O_4_ sensors were extracted from their DVP curves, specifically at potentials of 0.45, 0.4, and 0.35 V, respectively. The LOD values obtained were 0.013, 0.027, and 0.002 mM for the NiCo_2_O_4_, MnCo_2_O_4_, and Ni_0.5_Mn_0.5_Co_2_O_4_ sensors, respectively. Notably, the Ni_0.5_Mn_0.5_Co_2_O_4_ sensor exhibited the highest sensitivity and the lowest LOD among the three tested sensors, demonstrating its superior performance. Table 2 presents a comparative summary of key performance parameters for the electrochemical sensor fabricated using metal oxide-based nanostructures and their composites. As can be noticed, the Ni_0.5_Mn_0.5_Co_2_O_4_ sensor shows an impressive broad linear range, with LOD and sensitivity values on par with, or better than, those of leading sensors reported in the literature. Furthermore, the simple fabrication process and cost-effectiveness of the Ni_0.5_Mn_0.5_Co_2_O_4_ sensor make it a promising candidate for the large-scale production of electrochemical sensors.

**Table 2:** Comparison of the Acetaminophen Detection Performance of Electrochemical Sensors Fabricated Using Metal Oxide and CNT-Based Nanostructures^a^

**material of the electrochemical sensor**	**electrolyte solution**	**linear range**	**limit of detection**	**sensitivity**	**response time**	**av. lifetime**	**reference**
Co/Co_3_O_4_ nanoparticles and hollow nanoporous carbon	0.1 M PBS solution, pH 6	0.025–2.5 μM	0.0083 μM	152 μA cm^–2^ mM^–1^	-	-	(14)
β-cyclodextrin-functionalized reduced graphene oxide β-CD/RGO/GCE	0.1 M PBS solution, pH 7	10 μM–0.8 mM	2.3 μM	27.8 μA mM^–1^	240 s	-	(37)
Au@Fe_3_O_4_@rGO nanocomposite	0.1 M PBS solution, pH 7	0.01–0.28 μM	5 nM	1.57 μA cm^–2^ μM^–1^	93 s	-	(38)
NiO-GCE modified electrode	0.1 M PBS solution, pH 7	7.5–3000 μM	0.23 μM	91.0 mA cm^–2^ mM^–1^	-	-	(39)
CoFe_2_O_4_/graphite	0.1 M PBS solution, pH 6	80–200 μM	250 nM	0.065 μA cm^–2^ μM^–1^	-	-	(12)
TiO_2_ nanoparticles/carbon paste	0.2 M PBS, pH 7.4	10–70 μM	5.2 μM	-	-	-	(40)
multiwalled carbon nanotubes (MWCNTs) poly(amidoamine) dendrimers (G4.0 PAMAM)/GCE	0.2 M PBS, pH 7	0.7–200 μM	0.1 μM	4.0 μA cm^–2^ μM^–1^	-	-	(41)
MnCo_2_O_4_ with flexible graphene paper	0.1 M PBS solution, pH 7	4.64–1000 μM	1.39 μM	4.0 μA cm^–2^ μM^–1^	-	-	(15)
multiwalled carbon nanotubes (MWCNT) with polyarginine (polyArg)	0.05 M PBS, pH 7.4	1–80 μM	0.27 μM	0.061 μA μM^–1^	300 s	-	(42)
Ni_0.5_Mn_0.5_Co_2_O_4_	0.1 M PBS solution, pH 6.5	10–5000 μM	2.0 μM	38.2 μA cm^–2^ mM^–1^	35.4 s	0.31 s	this work

a The symbol “-” indicates data not available.

**Figure 7:**
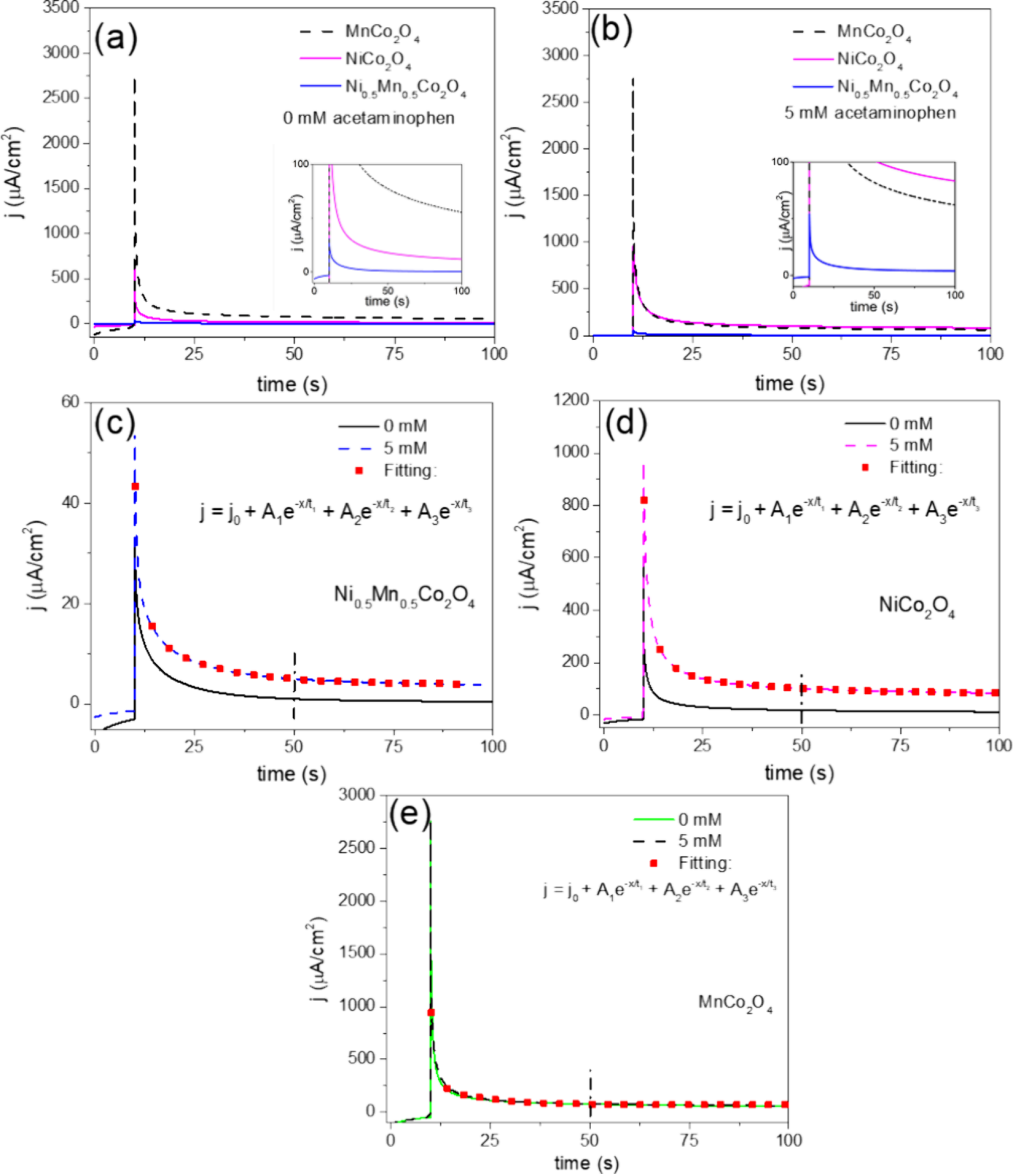
Chronoamperometric curves for the detection of acetaminophen at concentrations of (a) 0 mM and (b) 5.0 mM using different active materials on a carbon electrode. Panels (c), (d), and (e) display the chronoamperometric responses for Ni_0.5_Mn_0.5_Co_2_O_4_, NiCo_2_O_4_, and MnCo_2_O_4_, respectively, at 0 and 5.0 mM acetaminophen, along with their fittings to triple-exponential decay functions.

Figure 7 illustrates the current density responses of the three electrochemical sensors both in the absence (Figure 7a) and presence (Figure 7b) of acetaminophen in the electrolyte solution. As can be noticed, the Ni_0.5_Mn_0.5_Co_2_O_4_ sensor exhibits the fastest response in acetaminophen-free electrolytes compared to the other two sensors. Upon exposure to the electrolyte containing 5.0 mM acetaminophen, all sensors demonstrated a notable increase in current density, with the Ni_0.5_Mn_0.5_Co_2_O_4_ sensor maintaining the quickest response, followed closely by the NiCo_2_O_4_ sensor.

For a more detailed assessment of sensor performance, the current density responses of the fabricated sensors were recorded (Figures 7c–e). As can be noticed, both the Ni_0.5_Mn_0.5_Co_2_O_4_ and NiCo_2_O_4_ sensors exhibited a significant increase in current density at 5.0 mM acetaminophen concentration, while the MnCo_2_O_4_ sensor showed minimal variation between the presence and absence of the analyte. After 50 s, when the sensors reached saturation current density, the “percent signal change” was estimated using eq 8, where *j*_target_ and *j*_no_target_ are the current density responses for the analyte and no-analyte cases, respectively.^43,44^ The recorded percent signal changes were 355% for the Ni_0.5_Mn_0.5_Co_2_O_4_, 395% for the NiCo_2_O_4_, and 7.38% for the MnCo_2_O_4_ sensor, clearly demonstrating the superior performance of the Ni_0.5_Mn_0.5_Co_2_O_4_ and NiCo_2_O_4_ sensors.
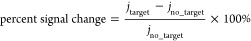
8

To evaluate the characteristic decay lifetimes of the current transients,^45^ a triple-exponential fit was applied to the chronoamperometric response curves using eq 9. The average decay lifetimes (τ_avg_)^46^ were then estimated using eq 10:
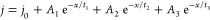
9
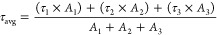
10

The fitting parameters and decay lifetimes are summarized in Table 3. The analysis revealed three distinct decay times (τ_1_, τ_2_, and τ_3_), each corresponding to a specific electrochemical kinetics process.^44^ The first decay time (τ_1_) is related to electron transfer at the electrode/electrolyte interface, and the second decay time (τ_2_) is linked to the diffusion-controlled transport of acetaminophen molecules from the bulk electrolyte to the electrode surface.^44^ Finally, the third decay time (τ_3_) can be related to pseudocapacitive effects arising from the intrinsic properties of the active material, such as its high surface area.^47^ Among the materials studied, the Ni_0.5_Mn_0.5_Co_2_O_4_ sensor exhibited shorter τ_1_ than other sensors, emphasizing its superior charge transfer kinetics. This performance is likely a result of its lower *R*_ct_. Meanwhile, the MnCo_2_O_4_ sensor displayed the longest τ_2_, consistent with its lower diffusion coefficient, which limits the transport of acetaminophen to the electrode surface. For τ_3_, the MnCo_2_O_4_ sensor demonstrated a faster decay time due to its larger surface area; however, this enhancement associated with pseudocapacitive effects was insufficient to improve the overall electrochemical sensor performance significantly. The results indicate that the Ni_0.5_Mn_0.5_Co_2_O_4_ sensor has the shortest average response lifetime of 0.31 s, reflecting a faster charge transport at the electrode/electrolyte interface than the other two sensors.^44,48^ Furthermore, the response time of the Ni_0.5_Mn_0.5_Co_2_O_4_ sensor, defined as the time required to reach 90% of the steady-state signal, was determined to be 35.4 s, which is significantly shorter than the response time of other electrochemical sensors reported for acetaminophen detection (Table 2). Additionally, the chronoamperometry curve presented in Figure S3 (Supporting Information) further demonstrates the high stability of the Ni_0.5_Mn_0.5_Co_2_O_4_ electrode over 10,000 s, in a 5 mM acetaminophen-containing 0.1 M PBS electrolyte solution. This remarkable stability further highlights the potential of the Ni_0.5_Mn_0.5_Co_2_O_4_ material for robust and reliable electrochemical sensing applications.

**Table 3:** Decay Lifetimes and Average Lifetimes of Chronoamperometric Responses for Different Electrochemical Sensors, as Determined by Triple-Exponential Decay Function Fitting

**electrode material**	**analyte conc.**	**τ**_**1**_**(s)**	**τ**_**2**_**(s)**	**τ**_**3**_**(s)**	***A***_**1**_	***A***_**2**_	***A***_**3**_	**av. lifetime (s)**
Ni_0.5_Mn_0.5_ Co_2_O_4_	5 mM	0.31	2.34	12.43	1.0 × 10^6^	0.26 × 10^–3^	17.6 × 10^–6^	0.31
NiCo_2_O_4_	5 mM	0.53	2.72	18.66	204.1	3.4 × 10^–3^	1.28 × 10^–4^	0.53
MnCo_2_O_4_	5 mM	8.74	8.74	0.82	0.00	0.00	3.41	0.82

**Figure 8:**
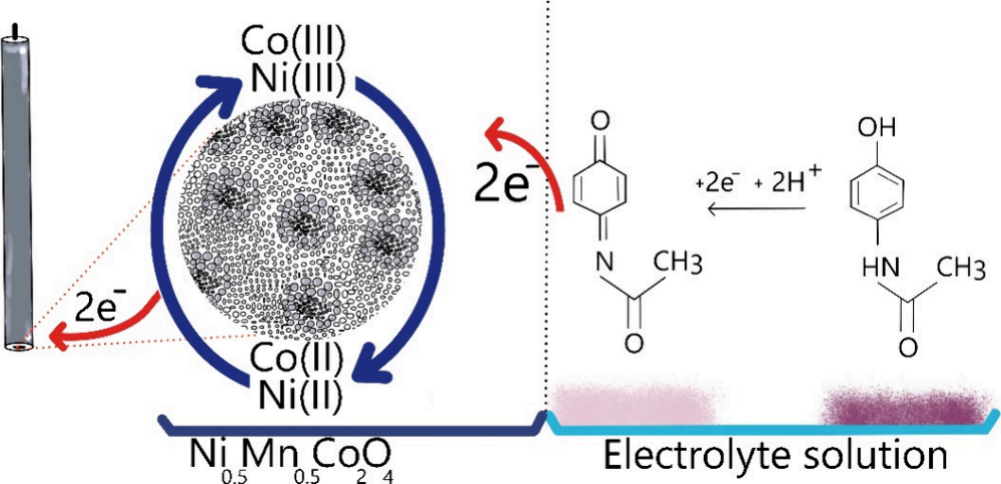
Schematic diagram illustrating the electro-oxidation process of acetaminophen using Ni_0.5_Mn_0.5_Co_2_O_4_ as the active material of the electrochemical sensor.

Figure 8 schematically illustrates the electrochemical sensing mechanism of acetaminophen using the MNCO sensors. The process begins with acetaminophen molecules migrating to the surface of the MNCO electrode under the influence of the applied electric field. Once the applied potential reaches the oxidation potential of acetaminophen, the molecules undergo electrochemical oxidation on the electrode surface, transforming into *N*-acetyl-4-benzoquinone imine (C_8_H_7_NO_2_).^49^ This electro-oxidation process releases two electrons and two protons, as shown in the following reaction (eq 11):^49,50^
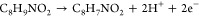
11

The released electrons are then transferred to the electron acceptor states of the transition metals present in the sensor material, specifically Co(III) and Ni(III).^51,52^ Since the 3d^6^ orbital of Co(III) is less stable than the 3d^7^ orbital of Co (II), an electron transitions from the 3d^6^ orbital to the 3d^7^ orbital, reducing Co(III) to Co(II). Similarly, an electron from the 3d^7^ orbital of Ni (III) is transferred to the 3d^8^ orbital, reducing Ni(III) to Ni(II).^14,53,54^ The overall reduction process can be represented as eq 12:
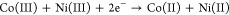
12

Consequently, the electrons in the 3d^8^ and 3d^7^ orbitals of Ni(II) and Co(II), respectively, are transferred to the π orbitals of the carbon electrode.^55^ This electron transfer restores the oxidation states of Ni(III) and Co(III), leading to the generation of a significant oxidation peak current for acetaminophen. Among the tested materials, the Ni_0.5_Mn_0.5_Co_2_O_4_ sensor exhibited a notably higher current increase compared to the NiCo_2_O_4_ and MnCo_2_O_4_ sensors. This is due to the presence of multiple active redox sites, specifically Co(III)/Co(II) and Ni(III)/Ni(II), which enhance its electrochemical performance. To demonstrate the multiples oxidation states in Ni_0.5_Mn_0.5_Co_2_O_4_, XPS analysis of all three samples is presented in Figure S4 (Supporting Information). The core-level Ni 2p spectra of Ni_0.5_Mn_0.5_Co_2_O_4_ and NiCo_2_O_4_ (Figure S4b,e) display characteristic Ni 2p_3/2_ and Ni 2p_1/2_ doublets, arising from spin–orbit splitting, at approximately 855 and 873 eV, respectively, along with shakeup satellite peaks at 860.6 and 872.96 eV. These features confirm the presence of Ni^2+^ and Ni^3+^ (O_h_ sites) in the spinel lattice.^16,56^ Similarly, the Co 2p spectra (Figure S4c,f,h) exhibit characteristic peaks at 779.07 and 794.3 eV, attributed to Co^3+^, and at 780.72 and 795.88 eV, corresponding to Co^2+^.^16,57^ A significant reduction in the peak area associated with Co^3+^ is observed in MnCo_2_O_4_, suggesting partial substitution of Co^3+^ by Mn^3+^ at O_h_ sites. Furthermore, the Mn 2p spectrum of MnCo_2_O_4_ (Figure 4g) exhibits peaks at 642 eV (Mn 2p_3/2_) and 653 eV (Mn 2p_1/2_).^16,57^ The deconvolution of peaks helped to identify the contributions from Mn^2+^ and Mn^3+^. Mn^3+^ ions predominantly occupy tetrahedral (*T*_d_) sites. Therefore, XPS analysis revealed features consistent with the better performance of the Ni_0.5_Mn_0.5_Co_2_O_4_ sensor, indicating the coexistence of Ni^2+^, Ni^3+^, Co^2+^, Co^3+^, and Mn^3+^ oxidation states. The study demonstrates the effects of mixed-metal cations in enhancing electrochemical detection. The superior performance of the MNCO sensor is attributed to the synergistic effects of its multiple oxidation states, optimum pore size, and reduced charge transfer resistance, all of which facilitate efficient electron transfer, thereby improving sensor sensitivity and response.

## 4 Conclusions

Using a simple, low-temperature gel-combustion technique, we successfully synthesized small and well-dispersed nickel/manganese mixed oxide nanoparticles with average sizes ranging from 10 to 26 nm. The electrochemical behavior of the nanoparticles was studied by employing them as active electrode materials in a three-electrode electrochemical cell, using neutral PBS as an electrolyte. The electrodes fabricated using these nanoparticles were tested for their ability to detect acetaminophen in PBS. The electrochemical sensor fabricated utilizing Ni_0.5_Mn_0.5_Co_2_O_4_ nanoparticles exhibited the best performance in detecting acetaminophen, achieving a sensitivity of 38.2 μA cm^–2^ mM^–1^ and a detection limit of 2.0 μM. The sensor’s low charge transfer resistance facilitated efficient electron transfer at the metal oxide/electrolyte interface, enhancing its overall electrochemical response. The sensor demonstrated a rapid chronoamperometric response with a decay lifetime of 0.31 s and response time of 35.4 s, highlighting its fast detection capability of acetaminophen. Moreover, the Ni_0.5_Mn_0.5_Co_2_O_4_ electrodes are stable over 10,000 s, in 5 mM acetaminophen-containing 0.1 M PBS electrolyte solution. Cyclic voltammetry analysis confirmed that the electron-mediated oxidation of acetaminophen governs the detection mechanism. The findings highlight the potential of Ni_0.5_Mn_0.5_Co_2_O_4_ nanoparticles for developing highly sensitive and efficient electrochemical sensors for monitoring pharmaceutical contaminants in water.

## Supporting Information Available

Size distribution histograms of different materials; magnification view of the main diffraction peak (311); table of EDS elemental composition; table of summary of *I*_pa_ and *E*_pa_ values obtained from CV curves for NiCo_2_O_4_ and MnCo_2_O_4_ electrodes; long-term chronoamperometric stability test curve for the Ni_0.5_Mn_0.5_Co_2_O_4_ sensor; XPS spectra of NiCo_2_O_4_, MnCo_2_O_4_, and Ni_0.5_Mn_0.5_Co_2_O_4_ nanoparticles (PDF)
