# Supplementary material for: Ni1–xMnxCo2O4 Nanoparticles as High-Performance Electrochemical Sensor Materials for Acetaminophen Monitoring
Source: ACS Omega. 2025 Mar 17;10(11):11250–63. doi: 10.1021/acsomega.4c10927 (PMC11947848; doi:10.1021/acsomega.4c10927)
Supplement: Supplementary file 1 — ao4c10927_si_001.pdf [file ao4c10927_si_001.pdf]

Supporting Information

$Ni_{1-x}Mn_xCo_2O_4$ Nanoparticles as High-performance Electrochemical Sensor Material for Acetaminophen Monitoring

Alba Arenas-Hernandez[1], Francisco Enrique Cancino-Gordillo[2], and Umapada Pal[1*]

[1]Institute of Physics, Autonomous University of Puebla, 18 Sur & Av. San Claudio, C.U., Puebla 72570, Mexico.

[2]Instituto de Energias Renovables, Universidad Nacional Autonoma de Mexico, Priv. Xochicalco S/N Temixco, Morelos 62580, Mexico.

*Corresponding author: E-mail: upal@ifuap.buap.mx

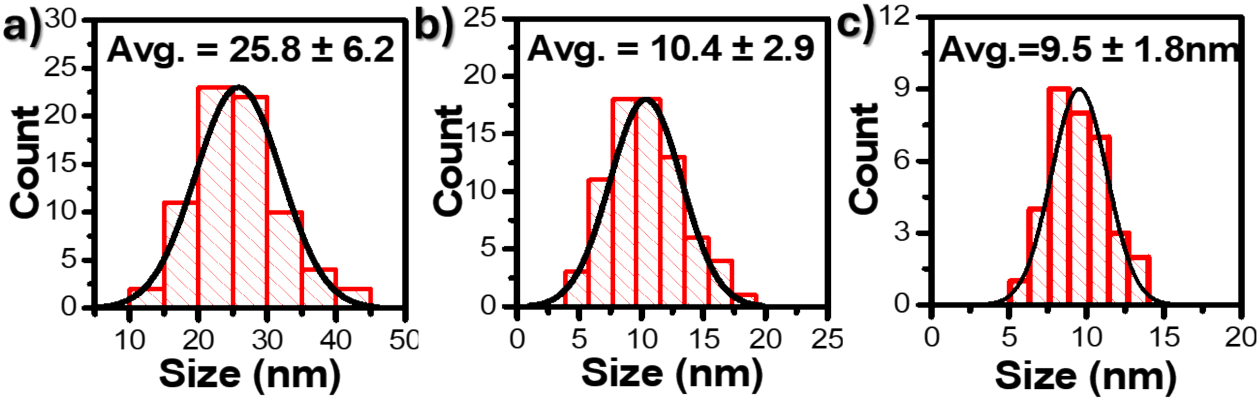

Figure S1. Size distribution histograms of the (a) $NiCo_2O_4$, (b) $Ni_{0.5}Mn_{0.5}Co_2O_4$, and (c) $MnCo_2O_4$.

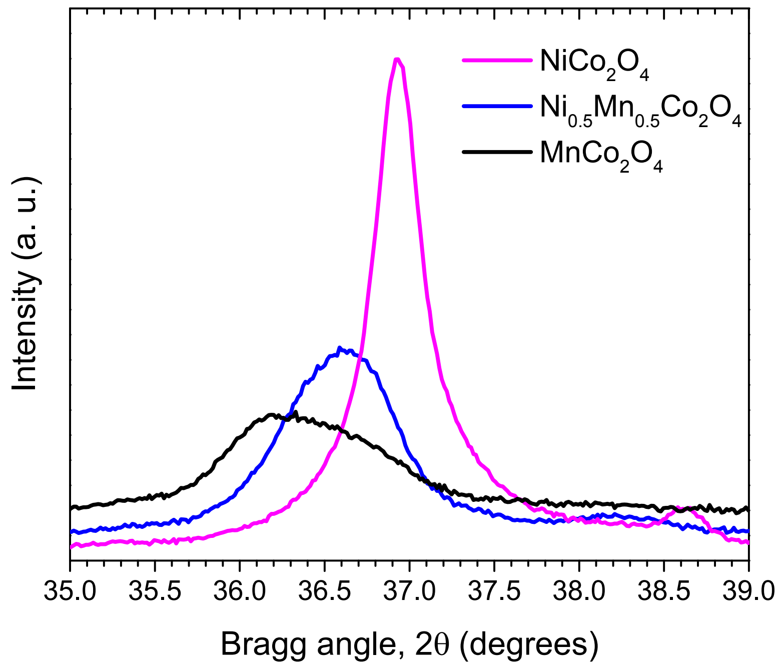

Figure S2. Magnification view of main diffraction peak (311) of the $NiCo_2O_4$, $Ni_{0.5}Mn_{0.5}Co_2O_4$, and $MnCo_2O_4$ materials.

Table S1. EDS estimated elemental composition of the synthesized $NiCo_2O_4$, $Ni_{0.5}Mn_{0.5}Co_2O_4$, and $MnCo_2O_4$ nanoparticles

Electrode material	***Mn (at %)***	***Ni (at %)***	***Co (at %)***	***O (at %)***	***C (at %)***
$MnCo_2O_4$	10.9 ± 1.0	0.0	23.5 ± 2.4	65.6 ± 3.4	11.32 ± 2.6
$NiCo_2O_4$	0.0	14.48 ± 1.0	28.1 ± 1.6	57.5 ± 2.3	15.12 ± 3.1
$Ni_{0.5}Mn_{0.5}Co_2O_4$	5.7 ± 0.6	6.6 ± 0.6	26.2 ± 2.3	61.6 ± 3.3	15.7 ± 2.7

Table S2. Summary of values I_{pa} and E_{pa}, obtained from CV curves for $NiCo_2O_4$ and $MnCo_2O_4$ electrodes for detecting 5 mM acetaminophen at different scan rates.

Material of the electrode	*Scan rate* (v)	I_{pa}	E_{pa}
$NiCo_2O_4$	25.0 mV/s	2.50×10^{-5} A	0.52 V
	12.5 mV/s	1.25×10^{-5} A	0.46 V
	10.0 mV/s	9.96×10^{-6} A	0.45 V
	7.5 mV/s	5.60×10^{-6} A	0.43 V
	5.0 mV/s	2.77×10^{-6} A	0.41 V
$MnCo_2O_4$	75.0 mV/s	2.84×10^{-5} A	0.56 V
	50.0 mV/s	2.53×10^{-5} A	0.53 V
	25.0 mV/s	1.59×10^{-5} A	0.48 V
	12.5 mV/s	1.04×10^{-5} A	0.43 V
	10.0 mV/s	8.21×10^{-6} A	0.42 V

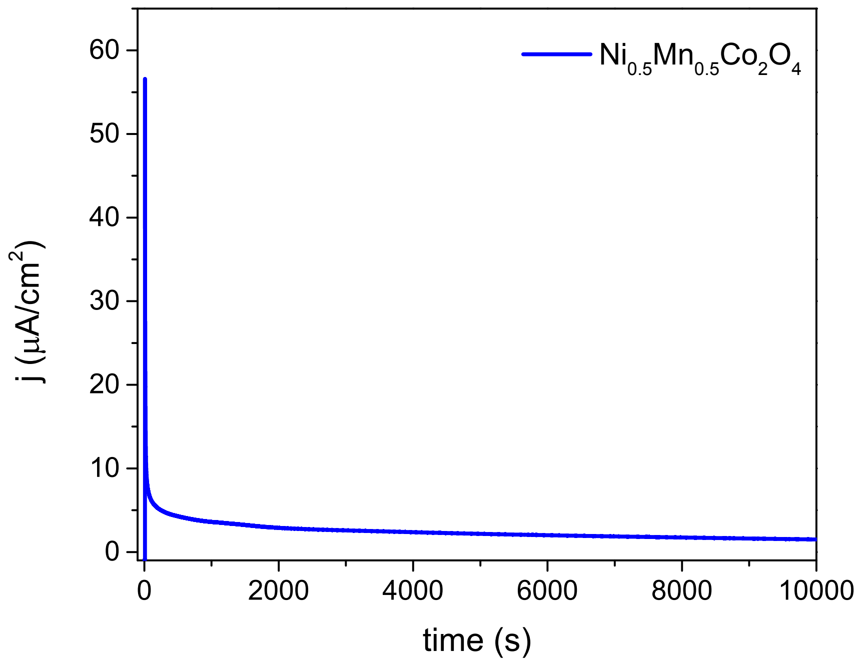

Figure S3. Long-term chronoamperometric stability test for the $Mn_{0.5}Ni_{0.5}Co_2O_4$ sensor with an acetaminophen concentration of 5 mM.

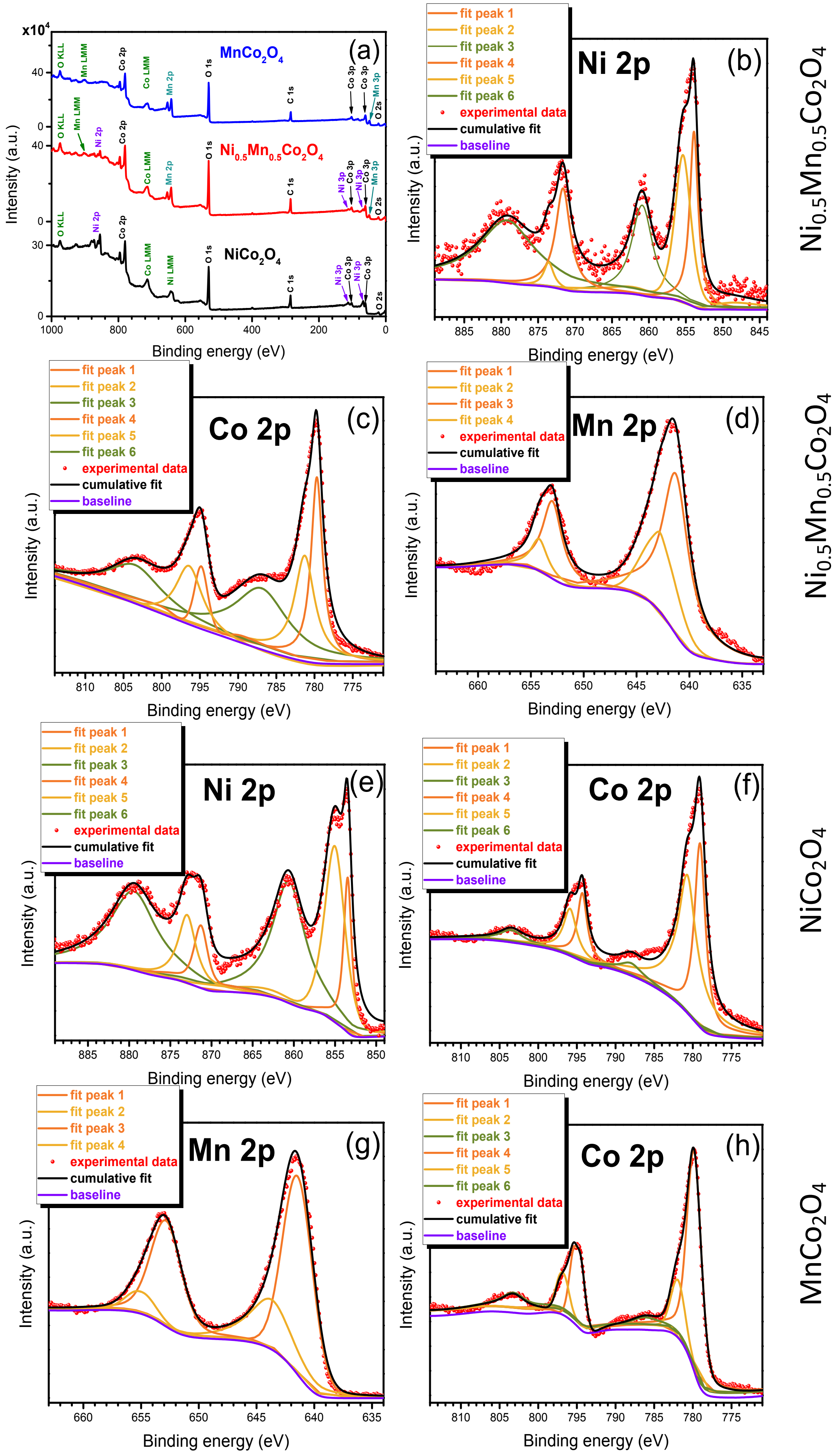

(a)
x10^4
MnCo2O4
Ni0.5Mn0.5Co2O4
NiCo2O4
O KLL
Mn LMM
Co 2p
Co LMM
Mn 2p
Ni 2p
Ni LMM
O 1s
C 1s
Co 3p
Ni 3p
Mn 3p
O 2s
Intensity (a.u.)
Binding energy (eV)
(b)
Ni 2p
(c)
Co 2p
(d)
Mn 2p
(e)
Ni 2p
(f)
Co 2p
(g)
Mn 2p
(h)
Co 2p
fit peak 1
fit peak 2
fit peak 3
fit peak 4
fit peak 5
fit peak 6
experimental data
cumulative fit
baseline
Ni0.5Mn0.5Co2O4
Ni0.5Mn0.5Co2O4
NiCo2O4
MnCo2O4

Figure S4. (a) Survey XPS spectra of $Ni_{1-x}Mn_xCo_2O_4$. High-resolution XPS spectra correspond to Co, Ni, and Mn orbitals in the (b,c,d) $Ni_{0.5}Mn_{0.5}Co_2O_4$, (e,f) $NiCo_2O_4$, and (g,h) $MnCo_2O_4$ nanoparticles.
